# Foot Musculature of the Common Marmoset (*Callithrix jacchus*): An Anatomical Study

**DOI:** 10.3390/ani16111659

**Published:** 2026-05-29

**Authors:** Robin D’Hooghe, Jolien Horemans, Jaco Bakker, Christophe Casteleyn

**Affiliations:** 1 Department of Morphology, Imaging, Orthopedics, Rehabilitation and Nutrition, Faculty of Veterinary Medicine, Ghent University, Salisburylaan 133, 9820 Merelbeke-Melle, Belgium; rodhoogh.dhooghe@ugent.be (R.D.); jolien.horemans@anicura.be (J.H.); 2 Anicura Dierenkliniek De Ark, Probastraat 1, 2235 Westmeerbeek, Belgium; 3 Animal Science Department, Biomedical Primate Research Centre, Lange Kleiweg, 161, 2288 GJ Rijswijk, The Netherlands; bakker@bprc.nl; 4 Department of Veterinary Sciences, Faculty of Pharmaceutical, Biomedical and Veterinary Sciences, University of Antwerp, Universiteitsplein 1, 2610 Wilrijk, Belgium

**Keywords:** common marmoset, anatomy, myology, foot, muscles, tendons

## Abstract

The common marmoset (*Callithrix jacchus*) is a New World monkey that was introduced as an animal model in biomedical research in the 1960s. In addition to their use in biomedical research, marmosets are also often housed in zoos. Their arboreal lifestyle requires a well-functioning foot. When confronted with foot injuries, the veterinarians responsible for the medical care of captive marmosets request easily available, clearly presented anatomical data. In addition, this anatomical data will be very valuable to gain a better understanding of marmosets’ locomotion. The scarce books and atlases on the anatomy of this species provide only line drawings, use archaic nomenclature, and/or exclude details on the foot. Color photographs were taken during the subsequent steps of the dissections of four common marmoset cadavers. Both dorsal and plantar views of the crural muscles with influence on the tarsus and foot, as well as the intrinsic foot musculature, are described in the guiding text. Contradictions with the available literature and a comparison with the rhesus monkey are included. A few remarkable differences with the rhesus monkey’s foot were observed. Furthermore, noteworthy additions to the existing literature are made. It is concluded that this work could serve as an anatomical basis for medical interventions on injured feet and for further locomotor studies.

## Simple Summary

The common marmoset (*Callithrix jacchus*) is a small monkey that is kept in captivity in zoos and research centers. Its foot, which is used for walking on thin branches and perches, can sometimes be injured. Consequently, the wounds must be tended by veterinarians who need knowledge of the anatomy of marmoset feet. In addition, this anatomical knowledge could be valuable for gaining a better understanding of their locomotion. Currently, this knowledge is lacking in the literature. To fulfill this gap, the muscles of the foot were examined by means of dissecting four marmoset cadavers. Color photographs are provided, which are described in the guiding text. Comparisons were made with the scarce literature on the common marmoset, the rhesus monkey, and humans. It was also noticed that the literature often used archaic terms, provided only line drawings, or failed to provide details on their feet. A few dissimilarities are observed when compared to the rhesus monkey.

## 1. Introduction

The white-tufted-ear or common marmoset (*Callithrix jacchus*) is a New World monkey that belongs to the Suborder *Haplorrhini*, Parvorder *Platyrrhini*, Family *Cebidae*, Subfamily *Callitrichinae* [1,2]. This non-human primate was introduced as an animal model in the 1960s and is now extensively used in biomedical research [3,4]. Research domains include neuroscience, reproductive biology, infectious disease, ethology, drug development, and safety assessment [4,5]. Its popularity can be traced back to its anatomical and physiological similarities with humans [6]. It often wins the competition with the rhesus monkey (*Macaca mulatta*), which is even more genetically related to man, on the basis of criteria like size, cost, husbandry, availability, ease of breeding in captivity, and biosafety issues [4,7,8].

Marmosets can be encountered in research facilities and zoos. Since the welfare of captive animals can be improved by enrichment programs, the housing facilities of common marmosets are typically provided with plenty of branches and ropes [8,9,10]. A well-functioning foot is pivotal to the welfare of the common marmoset as it is used for locomotion and arboreal support [11]. It is evident that the normal use of the foot can lead to lacerations of this body part. Unfortunately, injuries at the level of the extremities are often inflicted by aggression, e.g., when a new group member is introduced or when hierarchical disputes arise [12]. It is important that such scratching and biting wounds are tended by the veterinarians responsible for the medical care of the captive animals, as these can result in septicemia or endotoxemia [13]. Should these injuries at the level of the extremities, such as the fingers or the tail, fail to heal, amputation may be necessary [14]. It goes without saying that such interventions require a profound knowledge of the anatomy of the involved body parts, including the foot [11].

Comprehension of the marmoset’s hind limb and foot anatomy may also contribute to a better understanding of the locomotion and gait patterns in the marmoset. Marmosets express a convincing preference for asymmetrical gaits, such as gallops and bounds that present an aerial phase, over symmetrical gaits, such as walks and runs that lack such a phase. However, movements on narrow supports are typified by gaits lacking the aerial phase, and the narrower the support, the more the muscular grasping torque is produced by the hind limbs [15]. The ability to grasp objects is improved by the presence of friction ridges (dermatoglyphics) on the palms and soles of marmosets. Like humans, each individual has a unique palm- and soleprint. In addition, three inconstant flexion creases can be observed [16]. Another resemblance with humans is the manifestation of leading-limb preferences. The tendency for the right arm to be stronger is balanced by the tendency for the left leg to be stronger [17]. Locomotion on thin arboreal supports requires grip strength and clawless grasping hands and feet [18]. Surprisingly, *Callithrix jacchus* has a sharp claw (*tegula*) on each digit, with the exception of the hallux, which has a flat nail (*unguis*) and presents reduced pedal grasping [19]. As a result, they spend much time clinging to large trunks, showing the relationship between anatomy, behavior and ecology. Compared to humans, the common marmoset exhibits relatively smaller shoulder protractor, retractor, and abductor muscles, larger elbow extensor and rotator-cuff muscles in the forelimb, and smaller plantarflexor muscles in the hind limb [20].

Despite the ubiquitous use of the common marmoset in biomedical research, the number of currently available anatomical works on marmosets is very limited. The article “The anatomy of the common marmoset (*Hapale jacchus* Kuhl)” by Beattie [21] is considered a standard reference. It has, however, some shortcomings. As this work is almost 100 years old, the anatomical nomenclature, as well as the species name in the title, is obsolete in places. The illustrations that accompany the excellent textual descriptions of the anatomical structures consist of simplified black-and-white line drawings. Textual descriptions of the foot musculature are concise compared to those of the other body parts. Unfortunately, and surprisingly as well, any illustration of the foot is lacking. Another masterpiece in the field of primate anatomy was written by Diogo and Wood, who performed a phylogenetic analysis of primates based on the muscles of the head, neck, pectoral region and the upper limb [22]. One specimen of *Callithrix jacchus* was included, but no dissections of the hind limb and foot were executed. The standard reference by Beattie was recently revisited by Casteleyn and Bakker, who describe the anatomy of the common marmoset in a chapter of a book on the common marmoset in captivity and biomedical research [23]. With the focus on the biomedical researcher, no in-depth descriptions of the anatomy of the foot are provided. It is, however, much appreciated that the illustrations are color photographs taken during the dissections of several specimens.

It is the aim of the present study to fill a gap in the existing literature on the anatomy of the common marmoset, not only in terms of described body regions, in this case the hind limb and foot, but also in terms of mode of presentation, providing color photographs of dissected specimens. The gross anatomy of the muscles involved in the movements of the ankle (*tarsus*) and foot (*pes*) of the common marmoset is the focus. As extensively discussed above, these muscles have an immense functional value. The myology is systematically described, providing the origin, course, and insertion of each muscle. Multi-panel color photographs of dissected specimens accompany the textual descriptions. Both the intrinsic foot musculature, including the short muscles with origin and insertion at the level of the foot, affecting the mobility of the toes (*digiti pedis*), and the crural musculature with their long bellies situated at the crus (tibia and fibula), initiating the movements of the entire foot by influencing the ankle joint or specifically targeting the digital joints, are covered.

## 2. Materials and Methods

### 2.1. Animals

For this study, the biobank of the Biomedical Primate Research Center (BPRC), Rijswijk, The Netherlands (https://www.bprc.nl/bio-banken/bio-bank, accessed for the last time on 12 February 2026) was the source of four frozen (−20 °C) cadavers of the species *Callithrix jacchus*. The research included two adult male and two adult female captive-bred marmosets. All animals were between four and six years old and had a weight range between 350 and 400 g. The animals whose cadavers were examined were euthanized for reasons unrelated to the present study. These included welfare issues and research or diagnostics. No locomotor system abnormalities were noticed during the lifetimes of the living animals. Prior to euthanasia, the four animals were fasted for 16 h, but water always remained available. Immobilization of the animals was obtained by intramuscular injection of 12 mg/kg alphaxalone (Alfaxan Multidose^®^ 10 mg/mL, Jurox Limited, London, UK). Pentobarbital (60 mg/kg) (Euthasol^®^ 20%; AST Farma B.V., Oudewater, The Netherlands) was subsequently intravenously administered through the *vena saphena parva*. Two days in advance of the dissections, the frozen cadavers were thawed in a cold room (−6 °C), enabling the dissection of their pelvic limbs, in particular the crus and the foot.

### 2.2. Dissection

A total of eight hind limbs (both the left and right pelvic limbs of each of the four cadavers) were used in this study. They were first amputated and skinned, and the lower leg (crus) and foot were subsequently dissected from the superficial to the deepest layer. Both the cranial and caudal sides of the crus and the dorsal and plantar sides of the foot were examined. The observations made during the dissections of all eight hind limbs allowed for textually describing the common marmoset’s foot myology. However, the presented Figures all show the limbs from the left side, which is the conventionally illustrated side in most anatomic works. The dissections that were primarily performed by the naked eye were complemented by examinations under the stereomicroscope (Olympus SZX7, Olympus Belgium, Aartselaar, Belgium). The anatomical works on the common marmoset that were reviewed in the Introduction proved invaluable during the dissections [21,23]. Three works on the anatomy of the rhesus monkey, of which one specifically scrutinizes the musculature of the foot, were accessed in order to gain further insight into the primate foot [24,25,26]. Finally, a comparison was made with the human foot on the basis of two human anatomy atlases [27,28].

### 2.3. Imaging

A Canon EOS 450D body (Canon Inc., Tokyo, Japan) combined with a Canon EF-S 18–200 mm f/3.5–5.6 IS lens (Canon Inc.) was used to take macroscopic photographs. Stereomicroscopic images were obtained by a charge-coupled device camera (Olympus DP50, Olympus Belgium) that was mounted on a stereomicroscope (see above). The specimens were placed on a black background to avoid scattering the surrounding light while photographing. The software packages GIMP 2.10.30 (gimp.org) and Adobe Photoshop Elements 2025 (Adobe Inc., San Jose, CA, USA) were employed for cropping and equalizing the plain black background of the photographs. The lighting was adjusted, and the color temperature was optimized if necessary. Labeling of the selected, representative photographs and their arrangement in multi-panel figures was performed with Microsoft Office PowerPoint (Microsoft, Redmond, WA, USA).

### 2.4. Anatomical Terminology

The Nomina Anatomica Veterinaria (N.A.V.) [29] and the second edition of the Terminologia Anatomica (T.A.2) [30] were accessed to ensure that the labeled structures were correctly nominated. The veterinary anatomical terms derived from the N.A.V. were preferably used since the common marmoset is a non-human primate and not a human. Moreover, the wellbeing of captive common marmosets is supervised by veterinarians who would be familiar with veterinary and not human anatomy. It should, however, be respected that the N.A.V. does not list primate-specific terms, as it is restricted to domestic mammals. This implies that only structures that are shared by both the common marmoset and any of the domestic mammals included in the N.A.V. are listed. In other instances, analogous structures exhibited in the common marmoset and man were termed by means of the T.A.2. The Latin term is provided in italics between round brackets after the first mention of the anatomical structure in English. English terminology is exclusively applied in later descriptions. In contrast, only Latin terms are provided in the Figure legends.

## 3. Results

### 3.1. Crural Musculature

#### 3.1.1. Dorsal Approach

**Figure 1:**
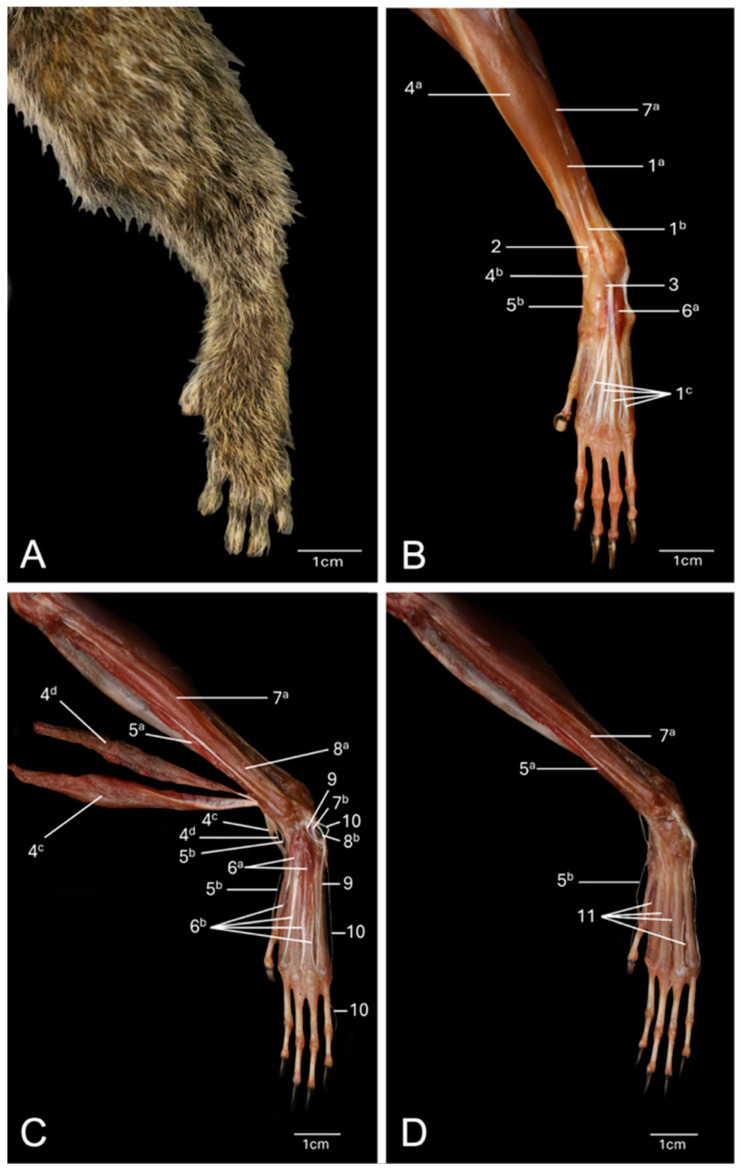
Dorsal view of the left crus and foot of the common marmoset: (**A**) Intact specimen showing the dense fur covering the entire dorsal side of the crus and foot; (**B**) Superficial muscle layer after the skin and the subcutaneous connective tissues are removed; (**C**) Deeper layer after the m. extensor digitorum longus is removed; (**D**) Deepest layer after removal of the de m. extensor digitorum et digiti primi/hallucis brevis. 1^a^: muscle belly of the m. extensor digitorum longus, 1^b^: transition of the muscle belly of the m. extensor digitorum longus into the common tendon, 1^c^: individual tendons of the m. extensor digitorum longus for the second to fifth digits, 2: retinaculum extensorum crurale, 3: retinaculum extensorum tarsale, 4^a^: muscle belly of the m. tibialis cranialis, 4^b^: presumed single tendon of the m. tibialis cranialis, 4^c^: m. tibialis cranialis medialis, 4^d^: m. tibialis cranialis lateralis, 5^a^: muscle belly of the m. extensor digiti primi/hallucis longus, 5^b^: tendon of the m. extensor digiti primi/hallucis longus, 6^a^: muscle belly of the m. extensor digitorum et digiti primi/hallucis brevis, 6^b^: inserting tendons of the m. extensor digitorum et digiti primi/hallucis brevis, 7^a^: muscle belly of the m. fibularis longus, 7^b^: inserting tendon of the m. fibularis longus, 8^a^: muscle belly of the m. fibularis brevis, 8^b^: inserting tendon of the m. fibularis brevis, 9: m. fibularis digiti quarti, 10: m. fibularis digiti quinti/minimi, and 11: mm. interossei pedis dorsalis.

The dorsal approach to the crural muscles that have long bellies situated at the level of the crus, and tendons that reach the level of the tarsus and the more distal metatarsus and digits, is presented in Figure 1. This crural musculature initiates the movements of the entire foot by influencing the ankle joint or specifically targeting the digital joints. The dorsal side of the crus and foot of *Callithrix jacchus* is completely covered with fur (Figure 1A).

The skin and the loose subcutaneous connective tissues, also known as the hypodermis or superficial fascia, are resected in Figure 1B. The superficial layer of the crural musculature can now be studied. The extensor digitorum longus muscle (*musculus (m.) extensor digitorum longus,* both in N.A.V. and T.A.2) (Figure 1B, no. 1^a^) is easily recognizable as a slender muscle with origin at the lateral tibial condyle, the crural interosseous membrane (*membrana interossea cruris*), and the fibula. The muscle belly passes into a long tendon (Figure 1B, no. 1^b^) at the level of the distal fourth of the crus. This tendon is covered by both the transverse crural ligament (*retinaculum extensorum crurale*/*retinaculum proximale*) (Figure 1B, no. 2), which is situated dorsally at the distal end of the crus where the malleoli can be found, and the sling-like tarsal ligament (*retinaculum extensorum tarsale*/*retinaculum distale*) (Figure 1B, no. 3), which is located more distal at the dorsal side of the tarsus. Both retinacula are reinforcements of the deep fascia at the dorsal side of the foot (*fascia dorsalis pedis*). They retain the position of the tendon during dorsiflexion of the foot. The common tendon splits into two tendons immediately distal to the distal retinaculum. Each of the resultant tendons presents a further bifurcation at the level of the proximal third of the third and fourth metatarsal bones (*os metatarsale III*/*tertium* and *IV*/*quartum*). As a consequence, four tiny tendons arise, which insert into the dorsal sides of the bases of the distal phalanges of the second to fifth digits (*digitus II*/*secundus*, *III*/*tertius*, *IV*/*quartus* and *V*/*quintus*) (Figure 1B, no. 1^c^). The extensor digitorum longus muscle thus extends the toes, except the hallux.

**Figure 2:**
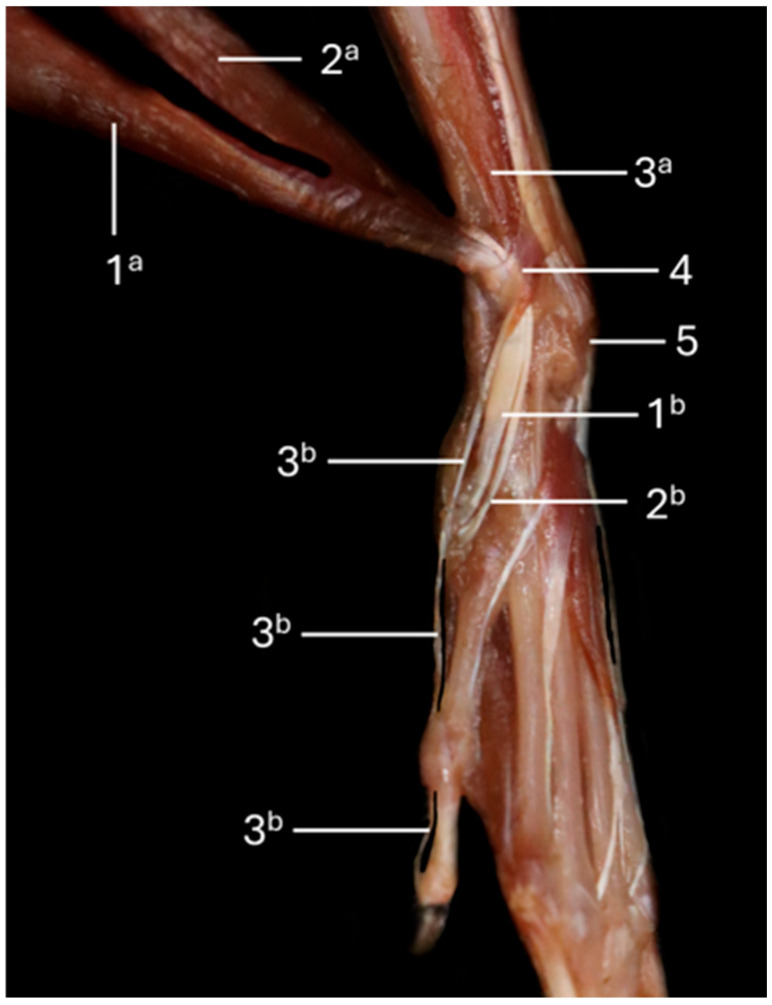
Medial view of the left tarsus and metatarsus of the common marmoset, emphasizing the inserting tendons of the medial and lateral bellies of the m. tibialis cranialis, and the tendon of the m. extensor digiti primi/hallucis longus. 1^a^: muscle belly of the m. tibialis cranialis medialis, 1^b^: tendon of the medial belly of the m. tibialis cranialis, 2^a^: muscle belly of the m. tibialis cranialis lateralis, 2^b^: tendon of the lateral belly of the m. tibialis cranialis, 3^a^: muscle belly of the m. extensor digiti primi/hallucis longus, 3^b^: tendon of the m. extensor digiti primi/hallucis longus, 4: retinaculum extensorum crurale, and 5: retinaculum extensorum tarsale.

Medial to the extensor digitorum longus muscle sits the prominent tibialis cranialis muscle (*m. tibialis cranialis* in N.A.V.) that originates at the lateral tibial condyle and the proximal two-thirds of the tibial diaphysis (*sulcus extensorum*) (Figure 1B, no. 4^a^). When its presumed tendon that runs over the medial side of the tarsus (Figure 1B, no. 4^b^) is further dissected, not a single tendon but a broader medial tendon (Figure 1C, no. 4^c^ and Figure 2, no. 1^a^ and 1^b^) and a thin lateral tendon (Figure 1C, no. 4^d^ and Figure 2, no. 2^a^ and 2^b^) can be recognized. Consequently, a medial and a lateral muscle belly can be identified when the respective tendons are followed in the proximal direction. The tendon of the medial belly attaches to the medial aspect of the first tarsal bone (*os tarsale I*/*primum* or *os cuneiforme mediale*), whereas the tendon of the lateral belly inserts into the head of the first metatarsal bone (*os metatarsale I*/*primum*). Inversion of the foot is attributed to the contraction of the medial muscle belly. The lateral muscle belly has the abduction of the hallux as its function.

The tendon of the extensor digiti primi/hallucis longus muscle (*m. extensor digiti primi longus* in the N.A.V./*m. extensor hallucis longus* in the T.A.2) lies immediately dorsolateral to the tendon of the lateral belly of the m. tibialis cranialis (Figure 2, no. 3^b^). When this tendon is followed in proximal direction (Figure 1B, no. 5^b^), the muscle belly that is formed at the level of the distal third of the crus cannot be traced as it is covered by the extensor digitorum longus and tibialis cranialis muscles. The mucle belly can only be followed over a longer distance once the extensor digitorum longus muscle is resected (Figure 1C, no. 5^a^). Now, the origin of this muscle, which is the crural interosseous membrane, can be recognized. When the tendon is followed in a distal direction, the insertion into the dorsal side of the terminal phalanx of the hallux is visible (Figure 1C, no. 5^b^). This muscle functions as the main extensor of the hallux. It additionally plays a supportive role in the dorsiflexion of the foot.

The resection of the extensor digitorum longus muscle enhances the visibility of the extensor digitorum et digiti primi/hallucis brevis muscle (*m. extensor digitorum et digiti primi*/*hallucis brevis* not in the N.A.V nor in the T.A.2, term borrowed from [21]). Its muscle belly was already visible on the superficial layer where it was partially obscured by the splitting tendon of the extensor digitorum longus muscle (Figure 1B, no. 6^a^). Once it is fully exposed (Figure 1C, no. 6^a^), the four diminutive muscle bellies that have their origins at the dorsolateral apsect of the calcaneal tuberosity (*tuber calcanei*) can be appreciated (Figure 1C, 6^b^). Each muscle belly sends a minuscule tendon to either the first, second, third, or fourth digit. The medial-most tendon attaches to the dorsolateral side of the proximal phalanx of the hallux, hereby supporting the function of the extensor digiti primi/hallucis longus muscle. The remaining three tendons insert into the dorsolateral sides of the distal phalanges of the second to fourth digits. As such, this muscle assists the extensor digitorum longus muscle in extending these digits.

**Figure 3:**
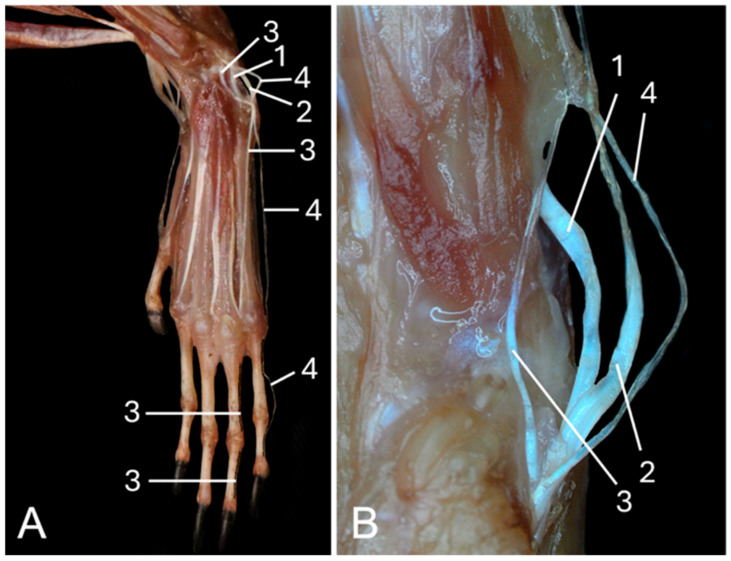
Dorsal view of the left foot of the common marmoset focusing on the inserting tendons of the fibularis complex: (**A**) Lower magnification; (**B**) Stereomicroscopic view of the lateral ankle region detailing the course and organization of the tendons. 1: m. fibularis longus, 2: m. fibularis brevis, 3: m. fibularis digiti quarti, and 4: m. fibularis digiti quinti/minimi.

The fibularis complex of the common marmoset consists of the fibularis longus muscle (*m. fibularis longus* both in N.A.V. and T.A.2), the fibularis brevis muscle (*m. fibularis brevis* both in N.A.V. and T.A.2), the fibularis digiti quarti muscle (*m. fibularis digiti quarti* not in the N.A.V. nor the T.A.2, term borrowed from [21]), and the fibularis digiti quinti/minimi muscle (*m. fibularis digiti quinti*/*minimi* not in the N.A.V. nor the T.A.2, term borrowed from [21]). The fibularis longus muscle (Figure 1B–D, no. 7^a^) originates at the head of the fibula (*caput fibulae*) and the proximal half of the fibular diaphysis. It initially runs lateral to the extensor digitorum longus muscle, leaves this muscle that continues its own course to the dorsal side of the tarsus, and finally passes the lateral malleolus with its tendon (Figure 1C, no. 7^b^ and Figure 3A,B, no. 1). At the level of the fourth tarsal bone (*os tarsale IV*/*quartum* or *os cuboideum*), this tendon bends to the plantar side of the foot. It runs deep against the plantar sides of the tarsal bones to insert into the abaxioplantar aspect of the basis of the first metatarsal bone. This allows for the eversion of the foot, extension of the ankle joint (plantar flexion), and opposition of the hallux. The fibularis brevis muscle (Figure 1C, no. 8^a^ and D) is initiated distal to the fibularis longus muscle, on the distal two-thirds of the fibular diaphysis. Its tendon (Figure 1C, no. 8^b^ and Figure 3A,B, no. 2) passes the lateral malleolus and runs lateral to the tendon of the fibularis longus muscle to attach to the tuberosity of the fifth metatarsal bone (*tuberositas ossis metatarsalis quinti*). Like the fibularis longus muscle, this muscle allows for the eversion and plantar flexion of the foot. The fibularis digiti quarti muscle (Figure 1C, no. 9 and Figure 3A,B, no. 3) and the fibularis digiti quinti/minimi muscle (Figure 1C, no. 10 and Figure 3A,B, no. 4) originate from the upper half of the caudolateral side of the fibula, deep to the fibularis longus muscle. The tendons run close to the tendon of the fibularis brevis muscle along its medial border. They insert into the abaxial side of the distal phalanx of the fourth and the fifth digit, respectively. These muscles assist in the extension of the fourth and fifth digits, which are mainly extended by the extensor digitorum longus muscle. In addition, both digits can be slightly abduced when the respective fibularis muscle contracts.

The dorsal interosseous muscles of the foot (*musculi (mm.) interossei pedis dorsales* in T.A.2) (Figure 1D, no. 11) are uncovered by the removal of the extensor digitorum et digiti primi/hallucis brevis muscle. They consist of four short muscles that are symmetrically positioned in the intermetatarsal spaces. They will be further elaborated on.

#### 3.1.2. Plantar Approach

**Figure 4:**
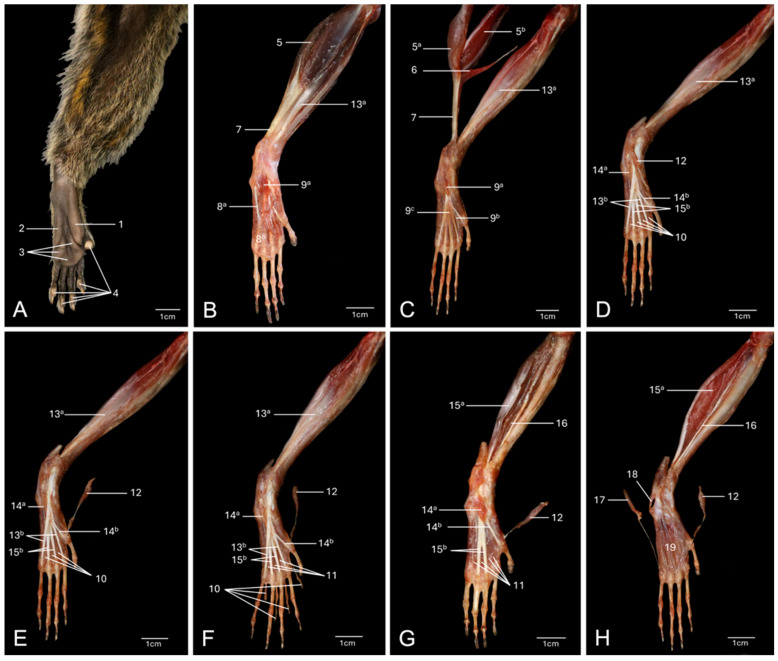
Plantar view of the left crus and foot of the common marmoset: (**A**) Intact specimen showing the dense fur covering the entire caudal side of the crus, but not the plantar side of the foot that is devoid of any hairs, enabling the observation of the thick epidermal covering that is characterized by the presence of dermatoglyphics; (**B**) Superficial muscle layer after the skin, the subcutaneous connective tissues, and the foot pads are removed; (**C**) Deeper layer after resecting the m. plantaris and transecting the origins of the gastrocnemius and soleus muscles (m. triceps surae) with their subsequent retraction; (**D**) Deeper layer after the resection of the m. flexor digitorum brevis caput superficiale and caput profundum, and the removal of the gastrocnemius and soleus muscles (m. triceps surae) by transecting the tendo Achilles; (**E**) Same level of dissection as the previous image but with transection of the origin of the m. abductor digiti primi/hallucis, which is retracted to the medial side of the foot; (**F**) Deeper layer after the mm. lumbricales pedis are removed; (**G**) Deeper layer subsequent to the resection of the m. flexor digitorum medialis/longus pars longa; (**H**) Deepest layer subsequent to the resection of the m. flexor digitorum medialis/longus pars brevis, the m. flexor digitorum lateralis, and after the transection of the origin of the m. abductor digiti quinti and its subsequent lateral retraction. 1: torus thenaris, 2: torus hypothenaris, 3: torus metatarsophalangeus, 4: torus apicalis, 5: m. gastrocnemius (5^a^: caput laterale, 5^b^: caput mediale), 6: m. soleus, 7: tendo Achilles, 8^a^: inserting tendon of the m. plantaris, 8^b^: aponeurosis plantaris, 9^a^: muscle belly of the m. flexor digitorum brevis caput superficiale, 9^b^: tendon of the m. flexor digitorum brevis caput superficiale, 9^c^: m. flexor digitorum brevis caput profundum, 10: mm. lumbricales pedis, 11: mm. contrahentes digitorum pedis, 12: m. abductor digiti primi/hallucis, 13^a^: belly of the m. flexor digitorum medialis/longus pars longa, 13^b^: individual tendons of the m. flexor digitorum medialis/longus pars longa, 14^a^: belly of the m. flexor digitorum medialis/longus pars brevis, 14^b^: inserting tendon of the m. flexor digitorum medialis/longus pars brevis, 15^a^: belly of the m. flexor digitorum lateralis, 15^b^: individual tendons of the m. flexor digitorum lateralis, 16: m. tibialis caudalis, 17: m. abductor digiti quinti/minimi, 18: m. abductor metatarsi quinti, and 19: mm. interossei pedis.

The plantar approach to the musculature of the crus is shown in Figure 4. In contrast to the dorsal side of the foot, the plantar side (*planta pedis*) is devoid of any hairs. The naked skin is characterized by the presence of dermatoglyphics. Four types of foot pads (*tori pedis* (singular: *torus pedis*)) can be recognized as they protrude by the presence of a subcutaneous fat cushion (*pulvinus*). The largest of the four is the elongated, broad thenar pad (*torus thenaris*) that is positioned on the thenar eminence (*eminentia thenaris*) at the basis of the hallux (Figure 4A, no. 1). The hypothenar pad (*torus hypothenaris*) that sits on the hypothenar eminence (*eminentia hypothenaris*) can be observed at the lateral side of the sole. It is also elongated but less broad compared to the thenar pad at its opposite side (Figure 4A, no. 2). Both are banana-shaped. The third type is the metatarsophalangeal pad (*torus metatarsophalangeus*), which is number three (Figure 4A, no. 3). The medial and lateral pads are located at the metatarsophalangeal joints of the second and fifth digits, respectively. They are pear-shaped. The middle can be considered an interdigital pad (*torus interdigitalis*), which is a triangular pad that is located at the bases of the third and fourth digits. Finally, an apical pad (*torus apicalis*) can be seen at the distal phalanx of each of the five toes (Figure 4A, no. 4).

Skinning the caudal side of the crus reveals the gastrocnemius muscle (*m. gastrocnemius* both in N.A.V. and T.A.2) (Figure 4B, no. 5), which is composed of a lateral head (*caput laterale*) (Figure 4C, no. 5^a^) and a medial head (*caput mediale*) (Figure 4C, no. 5^b^). These heads arise immediately proximal to the caudal aspects of the lateral and medial femoral condyles, respectively. The tendons of both muscle bellies join in the distal third of the crus to form a single tendon that inserts into the calcaneal tuber (*tuber calcanei*), allowing for the extension of the ankle joint (plantar flexion). After the origins of the gastrocnemius muscle are transected, both bellies can be retracted to visualize the soleus muscle (*m. soleus* both in N.A.V and T.A.2) that lies cranial (deep) to the gastrocnemius muscle (Figure 4C, no. 6). From the slender originating tendon that is attached to the caudal aspect of the fibular head (*caput fibulae*) arises a short muscle belly that fuses with the lateral head of the gastrocnemius muscle. As a consequence, the terminal, single tendon of the gastrocnemius muscle is reinforced and can be denominated as the calcaneal tendon (*tendo calcaneus*) or Achilles tendon (*tendo Achilles*) (Figure 4B,C, no. 7). The soleus muscle supports the action of the lateral and medial heads of the gastrocnemius muscle. The three muscle bellies form the triceps surae muscle (*m. triceps surae*).

Removing the skin further distal to the insertion of the Achilles tendon must be performed with caution in order not to damage the plantar fascia (*fascia plantaris*). This is the aponeurosis of the plantaris muscle (*m. plantaris* in T.A.2), which consists of connective tissue covering the intrinsic pedal muscles at the plantar side of the foot (Figure 4B, no. 8^a^). It is merged with the subcutaneous connective tissues underneath the metatarsophalangeal pads. When the plantar fascia is followed in the proximal direction, a small tendon arises at the lateral side of the sole of the foot (Figure 4B, no. 8^b^) that continues into a long, slender muscle running medially against the Achilles tendon. The origin of the plantaris muscle can be found at the caudal side of the femur, just proximal to the lateral condyle. It is, however, unfeasible to isolate the muscle belly of the plantaris muscle as it is merged with the lateral head of the gastrocnemius muscle. This muscle assists in the plantar flexion of the foot and assures the optimal pressure distribution in the sole.

The resection of the terminal tendon and fascia of the plantaris muscle uncovers the flexor digitorum brevis muscle (*m. flexor digitorum brevis*, both in N.A.V. and T.A.2). Its superficial head (*caput superficiale*) (Figure 4C, no. 9^a^) has origin on the caudomedial aspect of the calcaneal tuber and runs obliquely to the second digit. Here, a tiny tendon inserts into the plantar side of its second phalanx (Figure 4C, no. 9^b^). The deep head (*caput profundum*) (Figure 4C, no. 9^c^) originates more distally, in the middle of the metatarsus. Muscle fibers arise from the plantar sides of the individual tendons of the flexor digitorum medialis/longus muscle (*m. flexor digitorum medialis* in N.A.V./*m. flexor digitorum longus* in T.A.2) for digits I, II, and V, and from the plantar sides of the individual tendons of the flexor digitorum lateralis muscle (*m. flexor digitorum lateralis* in N.A.V.) to the third, fourth, and fifth digits. All these individual tendons of both muscles to the five digits are hereafter shortly called the “flexor tendons”. The weak muscle fibers of the deep head give origin to three tiny tendons that attach to the plantar sides of the bases of the second phalanges of the third, fourth, and fifth digits. Hence, all digits except the hallux receive a tendon from either the superficial or the deep head of the flexor digitorum brevis muscle, allowing for these digits to flex.

Both heads of the flexor digitorum brevis muscle are removed in Figure 4D. This allows for a better view of the above-defined “flexor tendons”. The four tiny, fusiform lumbrical muscles of the foot (*mm. lumbricales pedis* in T.A.2) (Figure 4D, no. 10) are located between the neighboring “flexor tendons”. They arise from the medial sides of the “flexor tendons” that run to digits II, III, IV, and V. They insert into the medial sides of the bases of the proximal phalanges of these digits. Multiple functions can be attributed to these muscles, i.e., extension of the digits and simultaneous flexion and adduction towards the hallux of the second to fifth digits by influencing their metatarsophalangeal joints. Once these four vermiform muscles are resected, the three contrahentes muscles of the foot (*mm. contrahentes digitorum pedis*, not in the N.A.V nor in the T.A.2, and term borrowed from [21]) (Figure 4F, no. 11) can be examined. These arise with a common aponeurosis from the tendon of the fibularis longus muscle that runs deep against the plantar sides of the tarsal bones. The three contrahentes muscles insert into the axial sides of the proximal phalanges of the second, fourth, and fifth digits. Their function lies in the adduction and slight flexion of digits II, IV, and V. The contrahens muscles of the foot are elaborated below.

**Figure 5:**
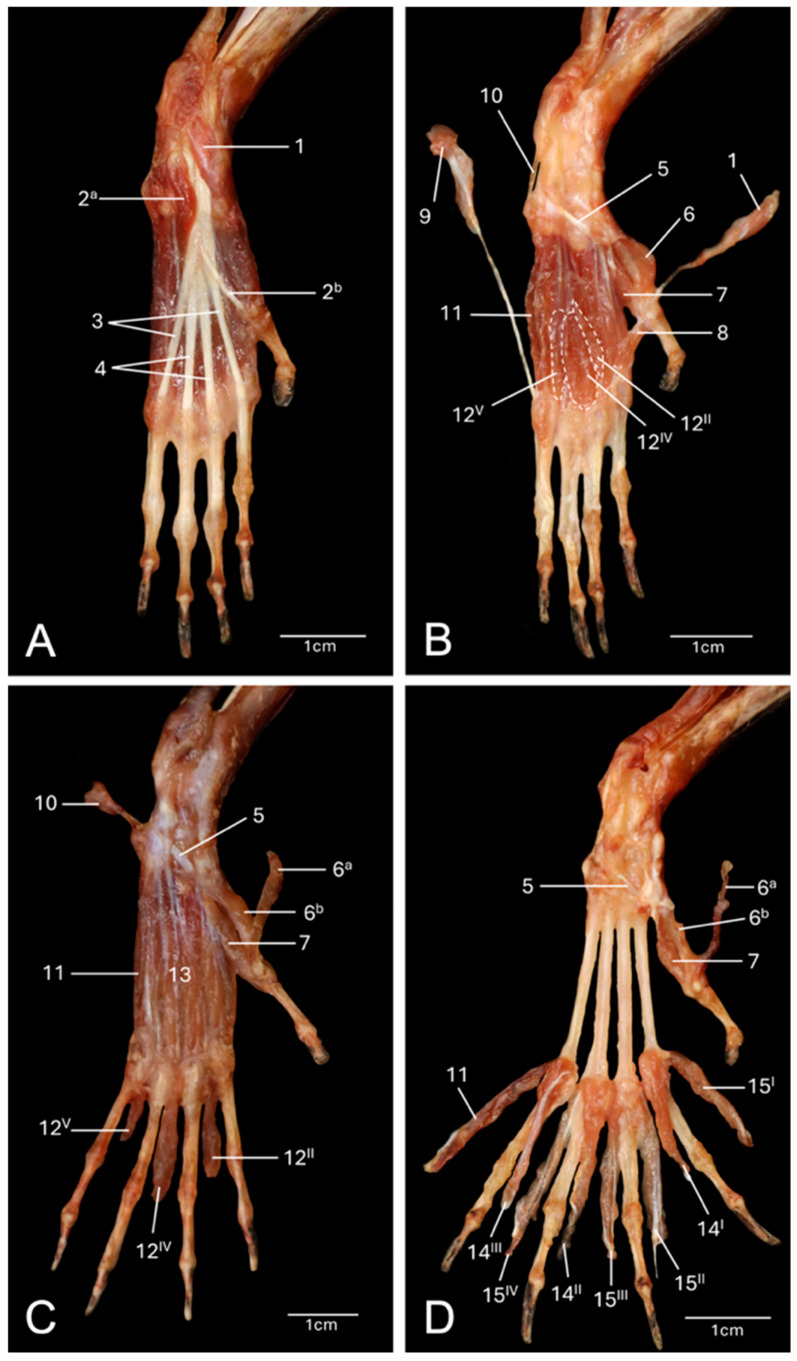
Plantar view of the left foot of the common marmoset: (**A**) Superficial muscle layer after the removal of the m. plantaris, m. flexor digitorum brevis and mm. lumbricales pedis; (**B**) Deeper layer after the subsequent resection of the m. flexor digitorum medialis/longus pars brevis and pars longa, m. flexor digitorum lateralis, and reflection of the m. abductor digiti primi/hallucis and m. abductor digiti quinti/minimi; (**C**) Deepest layer after the m. abductor digiti primi/hallucis and m. abductor digiti quinti/minimi are removed, the origins of the mm. contrahentes digitorum pedis are transected, and the muscles are retracted in the distal direction, and subsequent to the retraction of the m. abductor metatarsi quinti and the superficial part of the medial head of the m. flexor digiti primi/hallucis brevis; (**D**) Plantar view of the second to fifth metatarsal bones after the origins of the mm. interossei plantares and mm. interossei pedis dorsales are detached and the muscles are retracted in a distal direction towards their insertions. 1: m. abductor digiti primi/hallucis, 2^a^: muscle belly of m. flexor digitorum medialis/longus pars brevis, 2^b^: inserting tendon of m. flexor digitorum medialis/longus pars brevis, 3: inserting tendons of m. flexor digitorum medialis/longus pars longa, 4: inserting tendons of m. flexor digitorum lateralis, 5: inserting tendon of the m. fibularis longus, 6: m. flexor digiti primi/hallucis brevis caput mediale (6^a^: pars superficialis, 6^b^: pars profunda), 7: m. flexor digiti primi/hallucis brevis caput laterale, 8: m. adductor digiti primi/hallucis caput transversum, 9: m. abductor digiti quinti/minimi, 10: m. abductor metatarsi quinti, 11: m. flexor digiti quinti/minimi (brevis), 12^II^: m. contrahens digiti II/secundi pedis, 12^IV^: m. contrahens digiti IV/quarti pedis, 12^V^: m. contrahens digiti V/quinti pedis, 13: mm. interossei pedis, 14^I^: m. interosseus pedis plantaris I/primus, 14^II^: m. interosseus pedis plantaris II/secundus, 14^III^: m. interosseus pedis plantaris III/tertius, 15^I^: m. interosseus pedis dorsalis I/primus, 15^II^: m. interosseus pedis dorsalis II/secundus, 15^III^: m. interosseus pedis dorsalis III/tertius, and 15^IV^: m. interosseus pedis dorsalis IV/quartus.

The abductor digiti primi/hallucis muscle (*m. abductor digiti primi* in the N.A.V./*m. abductor hallucis* in the T.A.2) can also be observed in Figure 4D (no. 12). This short muscle runs obliquely from the medioplantar aspect of the calcaneal tuber, crosses the plantar side of the combined common tendons of the flexor digitorum medialis/longus and flexor digitorum lateralis muscles, and finally attaches to the abaxial side of the proximal phalanx of the hallux. Contraction of this muscle leads to abduction of the hallux. This intrinsic foot muscle is scrutinized below by means of Figure 5.

The combined common tendons of the flexor digitorum medialis/longus and flexor digitorum lateralis muscles, traveling along the sustentaculum of the talus, can be scrutinized in Figure 4E, after the resection of the abductor digiti primi/hallucis muscle. The flexor digitorum medialis/longus muscle is complex in *Callithrix jacchus* as it consists of a long part (*pars longa*) and a short part (*pars brevis*). When the common tendon of the long part is followed in the proximal direction, a muscle belly (Figure 4E, no. 13^a^) arises at the medial border of the medial head of the gastrocnemius muscle. Its origin is located at the caudal side of the middle third of the tibia. When this common tendon is followed in a distal direction, it is observed that it splits into two at the proximal aspect of the metatarsus. The arisen two individual tendons for digits II and V (Figure 4E, no. 13^b^) attach to the plantar sides of their distal phalanges. The consequence of contraction of the long part is flexion of both digits, but also extension of the ankle. The short part has an individual, rectangular muscle belly (Figure 4E, no. 14^a^) that originates from the lateroplantar side of the calcaneus. The muscle belly subsequently travels obliquely between the individual tendons of the flexor digitorum medialis/longus muscle for digits II and V towards the hallux. The short head enables the flexion of the hallux, as its relatively long tendon inserts into the plantar side of its distal phalanx. It is summarized that the flexor digitorum medialis/longus muscle sends tendons to digits I, II, and V to flex these.

The common tendon of the flexor digitorum lateralis muscle also lies dorsal and is thus covered by the common tendon of the flexor digitorum medialis/longus muscle, where they pass the sustentaculum of the talus. The latter muscle is removed in Figure 4G. As a result, the common tendon of the flexor digitorum lateralis muscle can easily be followed in both proximal and distal directions. Proximally, a unipennate muscle belly (Figure 4G, no. 15^a^) arises from the common tendon. Its origin is the mediocaudal aspect of the fibula and the crural interosseous membrane. The common tendon splits into two individual tendons (Figure 4G, no. 15^b^) at the middle of the metatarsus. Since they insert into the plantar sides of the distal phalanges of digits III and IV, the flexor digitorum lateralis muscle flexes both digits. It is summarized that the flexor digitorum medialis/longus and flexor digitorum lateralis muscles act together to flex each of the five digits.

The tibialis caudalis muscle (*m. tibialis caudalis*, both in N.A.V. and T.A.2) (Figure 4G, no. 16) is fully exposed in Figure 4F, after the removal of the flexor digitorum lateralis muscle. The slender muscle belly that is located medial to the muscle belly of the flexor digitorum lateralis muscle starts at the proximal half of the caudal side of the tibia, the crural interosseous membrane, and the medial side of the fibula. The long tendon also passes the sustentaculum of the talus and finally attaches to the plantar side of the bases of the second, third, and fourth metatarsal bones. The muscle aids in plantar flexion and enables the inversion of the foot.

The abductor digiti quinti/minimi muscle (*m. abductor digiti quinti* in the N.A.V./*m. abductor digiti minimi manus* in the T.A.2) can also be discerned in Figure 4H (no. 17). The slender muscle belly has its origin on the calcaneal tuber, just lateral to the origin of the abductor hallucis/digiti primi muscle. Its long tendon inserts into the abaxial side of the proximal phalanx of the fifth digit. The abductor metatarsi quinti muscle (*m. abductor metatarsi quinti* in the T.A.2) (Figure 4H, no. 18) also starts from the calcaneal tuber. The short muscle belly sends a thin tendon to the tuberosity of the fifth metatarsal bone. Both intrinsic foot muscles that abduce the fifth digit are elaborated below by means of Figure 5.

Finally, the interosseous muscles of the foot (*mm. interossei* in N.A.V.) are mentioned here (Figure 4H, no. 19). As will be elaborated on below using Figure 5 and Table 1, they are composed of three plantar and four dorsal interosseous muscles. The plantar interosseous muscles (*mm. interossei plantares*) arise from the plantar side of the second metatarsal bone (*os metatarsale II*/*secundum*), the fourth metatarsal bone, and the fifth metatarsal bone (*os metatarsale V*/*quintum*). The dorsal interosseous muscles of the foot are touched upon above. The contrahentes muscles of the foot that were mentioned above and labeled as no. 11 in Figure 4F are closely associated with the interosseous muscles of the foot. Therefore, these muscles cannot be individually identified in Figure 4F. Figure 5 is more suitable in this regard.

### 3.2. Intrinsic Foot Musculature

#### 3.2.1. Muscles of the Hallux

The intrinsic musculature of the foot consists of all the short muscles that have their origins and insertions at the level of the foot. The muscles that influence the hallux can be examined after resecting the plantaris muscle, the flexor digitorum brevis muscle, and the lumbrical muscles of the foot. The abductor digiti primi/hallucis muscle (Figure 5A, no. 1) and the short part of the flexor digitorum medialis/longus muscle with its rectangular muscle belly (Figure 5A, no. 2^a^) and relatively long tendon (Figure 5A, no. 2^b^) were already discussed above. It is recapitulated here that the abductor digiti primi/hallucis muscle inserts into the abaxial side of the proximal phalanx of the hallux, whereas the short head of the flexor digitorum medialis/longus muscle ends at the plantar side of the distal hallucal phalanx. Figure 5A also shows the two individual tendons of the long part of the flexor digitorum medialis/longus muscle (Figure 5A, no. 3) to digits II and V, and the two individual tendons of the *flexor digitorum lateralis muscle* (Figure 5A, no. 4) to digits III and IV.

Figure 5B presents a view after the origin of the abductor digiti primi/hallucis muscle is detached from the medioplantar aspect of the calcaneal tuber, and both the short and long parts of the flexor digitorum medialis/longus muscle and the flexor digitorum lateralis muscle are resected. Now, the course of the inserting tendon of the fibularis longus muscle (Figure 5B, no. 5), which was previously discussed in the chapter on the dorsal approach to the crural musculature, can be followed. The insertion of the tendon into the abaxioplantar aspect of the basis of the first metatarsal bone is, however, still obscured by the origins of the flexor digiti primi/hallucis brevis muscle (*m. flexor digiti primi brevis* in N.A.V./*m. flexor hallucis brevis* in T.A.2). This muscle has indeed several origins as it is composed of a medial head (Figure 5B, no. 6) and a lateral head (Figure 5B, no. 7). Further complicating this matter, the medial head consists of a superficial part (*pars superficialis*) (Figure 5C, no. 6^a^) and a deep part (*pars profunda*) (Figure 5C, no. 6^b^), which both start at the first tarsal bone alias medial cuneiform bone (*os tarsale primum* alias *os cuneiforme mediale*) and the central tarsal bone alias navicular bone (*os tarsi centrale* alias *os naviculare*). Both parts of the medial head insert together with the tendon of the abductor digiti primi/hallucis muscle into the abaxial aspect of the proximal phalanx of the hallux. The lateral head finds its origin on the first tarsal bone and attaches to the axial side of the proximal hallucal phalanx. Both heads realize flexion of the hallux.

Returning to Figure 5B allows for the further study of the intrinsic musculature of the hallux. Only the transverse head (*caput transversum*) of the adductor digiti primi/hallucis muscle (*m. adductor digiti primi* in the N.A.V./*m. adductor hallucis* in the T.A.2) could be recognized in the dissected specimens (Figure 5B, no. 9). This muscle has its origin on the heads of the second and third metatarsal bones as well as the contrahentes II and IV muscles that are scrutinized below. Insertion is into the axial side of the first metatarsal bone and the proximal phalanx of the hallux. Opposition and adduction of the hallux is the produced action of this muscle.

#### 3.2.2. Muscles of the Fifth Digit

The intrinsic musculature of the fifth digit consists of three muscles. The abductor digiti quinti/minimi muscle (*m. abductor digiti quinti*/*minimi*) (Figure 5A, no. 9) has a short, rectangular muscle belly that originates from the calcaneal tuber, just lateral to the origin of the abductor digiti primi/hallucis muscle. Its long tendon ends at the abaxial side of the proximal phalanx of the fifth digit. The second intrinsic muscle of the fifth digit is the short and tiny abductor metatarsi quinti muscle (Figure 5B, no. 10) that also starts from the calcaneal tuber. The thin tendon inserts into the tuberosity of the fifth metatarsal bone. The abductor metatarsi quinti muscle is situated lateral to the abductor digiti quinti/minimi muscle. Both muscles abduce the fifth digit. Finally, the flexor digiti quinti/minimi (brevis) muscle (*m. flexor digiti quinti* in N.A.V./*m. flexor digiti minimi (brevis)* in T.A.2) (Figure 5B, no. 11) is considered. The distal aspects of both the fifth metatarsal bone and the tendon of the fibularis longus muscle are the origins of this long, slim muscle. By its attachment to the abaxial side of the proximal phalanx of the fifth digit, it enables the flexion of this digit.

#### 3.2.3. Deep Plantar Musculature

The musculature located deep in the sole of the foot, lying against the metatarsus, is composed of the contrahentes muscles of the foot and the interosseous muscles of the foot. These muscles can be studied from Figure 5B onwards, after the removal of the tendons of the flexor digitorum medialis/longus muscle and the flexor digitorum lateralis muscle that run plantar to and thus highly obscure these. The fusiform contrahentes muscles of the foot are triple. They are situated in the middle of the sole, plantar to the interosseous muscles of the foot. They have a common origin in the form of an aponeurosis that attaches to the tendon of the fibularis longus muscle, where it runs deep against the plantar sides of the tarsal bones. The contrahens muscle of the second digit (*m. contrahens digiti II*/*secundi pedis*) (Figure 5B, no. 12^II^) is the most medial of the three, attaching to the axial side of the basis of the proximal phalanx of the second digit. The contrahens muscle of the fourth digit (*m. contrahens digiti IV*/*quarti pedis*) (Figure 5B, no. 12^IV^) sits in the middle, presenting its insertion into the axial aspect of the basis of the proximal phalanx of the fourth digit. Finally, the more lateral contrahens muscle of the fifth digit (*m. contrahens digiti V*/*quinti pedis*) (Figure 5B, no. 12^V^) inserts into the axial side of the proximal phalanx of the fifth digit. The contraction of the contrahentes muscles of the foot results in the adduction and modest flexion of the second, fourth, and fifth digits.

The interosseous muscles of the foot (Figure 5C, no. 13) are positioned deep to the contrahentes muscles of the foot. Transecting the origins of the latter muscles and retracting them in a distal direction enables the study of the seven interosseous muscles of the foot, i.e., three that can only be observed from the plantar side of the metatarsus and four that can also be seen from the dorsal side. The adjectives in the names of the interosseous muscles, whether “plantaris” or “dorsalis”, refer to this statement. The three plantar interosseous muscles of the foot, i.e., the first plantar interosseous muscle (*m. interosseus pedis plantaris primus*) (Figure 5D, no. 14^I^)*,* the second plantar interosseous muscle (*m. interosseus pedis plantaris secundus*) (Figure 5D, no. 14^II^), and the third plantar interosseous muscle (*m. interosseus pedis plantaris tertius*) (Figure 5D, no. 14^III^), originate at the plantar sides of the second, fourth, or fifth metatarsal bones. Each of the four dorsal interosseous muscles of the foot is located in one of the four intermetatarsal spaces *(spatia interossea metatarsi).* The number of the dorsal interosseous muscle refers to the number of the intermetatarsal space it sits in. They are bipennate muscles, which means that they have a lateral attachment to the medially located metatarsal bone and a medial attachment to the laterally located metatarsal bone of the respective intermetatarsal space.

Based on the origin, the interosseous muscles of the foot can be clustered into a medial and a lateral group. The medial group consists of the first plantar interosseous muscle, the first dorsal interosseous muscle (*m. interosseus pedis dorsalis primus*) (Figure 5D, no. 15^I^), and the second dorsal interosseous muscle (*m. interosseus pedis dorsalis secundus*) (Figure 5D, no. 15^II^). These all have a common origin at the plantar aspect of the base of the second metatarsal bone. The lateral group is composed of the second and third plantar interosseous muscles, the third dorsal interosseous muscle (*m. interosseus pedis dorsalis tertius*) (Figure 5D, no. 15^III^), and the fourth dorsal interosseous muscle (*m. interosseus pedis dorsalis quartus*) (Figure 5D, no. 15^IV^). These all have origins on the plantar sides of the fourth and fifth metatarsal bones. An overview of the origin, insertion, and function of the seven interosseous muscles of the foot is given in Table 1.

An overview of all the described muscles with their origins, insertions, and modes of action can be found in Table 2.

## 4. Discussion

This study provides a full elaboration on the anatomy of the musculature of the lower hind limb and the foot of the common marmoset. The rationale of this study is deduced from the fact that the literature on the foot musculature of the common marmoset is inadequate. This is surprising since pedal muscles play pivotal roles in the mobility and, hence, the welfare of the animal. The lack of specific anatomical data on the foot musculature of the common marmoset forced the investigators to seek guidance during the dissections, primarily in publications on the anatomy of the rhesus monkey. The origins, topography, and insertions of the observed muscles were systematically compared with the descriptions of the rhesus monkey pedal muscles. In particular, the recently published study entitled “The Foot Musculature of the Rhesus Monkey (*Macaca mulatta*): An Anatomical Study” was invaluable [26]. This work revisited the anatomy of the foot musculature of the rhesus monkey that was described in previously published atlases [24,25]. Since differences between the anatomy of the common marmoset, which is a New World monkey, and the rhesus monkey, which is an Old World monkey, were expected [22], comparison was also made with humans [27,28] and domestic mammals [31] in order to obtain a correct identification of the dissected crural and pedal muscles. Not only are the observed muscles described in the main text, but they are also depicted in several multi-panel figures that represent the subsequent steps of the dissection, working from the superficial layer towards the deepest layer of the crural and pedal musculature. As a result of this approach, the present work may serve as an anatomical atlas or dissection guide. In combination with the use of veterinary anatomical nomenclature, veterinarians charged with the medical follow-up of captive common marmosets (e.g., research facilities or zoos) will not go unprepared into wound management and surgical interventions. It was, however, not always possible to apply the veterinary anatomical terminology that is listed in the N.A.V. [29] since the common marmoset does not belong to the species that are included in this work, i.e., rabbits, cats, dogs, horses, cattle, sheep, goats, and pigs. Hence, the nomenclature for muscles that are specific for primates was derived from the available literature on the anatomy of the common marmoset [21,23], the rhesus monkey [24,25,26], or the T.A.2 [30].

The musculature located at the dorsal side of the crus and foot is largely described previously [21,23]. However, two gaps are noticed in the literature. Although undoubtedly identified during the dissections, the extensor digitorum et digiti primi/hallucis brevis muscle has not been reported yet in the common marmoset. This muscle has an analog in the rhesus monkey [24,25,26] but not in man [27,28,30].

Secondly, the dissections revealed that the fibularis complex in the common marmoset is composed of four fibularis muscles. The consulted literature on the foot musculature of *Callithrix jacchus* [21,23] and *Macaca mulatta* [24,25,26] only mentions the presence of the fibularis longus, fibularis brevis, and fibularis digiti quinti/minimi muscles in these species. Stereomicroscopic dissections enabled the identification of a fourth fibularis muscle in the common marmoset, i.e., the fibularis digiti quarti muscle. This muscle is present in a minority of humans [32] and is also described in rodents [33]. The fibularis digiti quinti/minimi muscle can also be present in humans [34]. Furthermore, the common marmoset and humans consistently have the fibularis longus and fibularis brevis muscles in common. Nevertheless, the fibularis complex is differently composed in the common marmoset and humans since the latter species most often also presents the fibularis tertius muscle. This muscle is rarely found in primates [35]. Nonetheless, it is a typical trait of ungulates, including horses, oxen, and small ruminants [31].

Far more discrepancies are noted between the existing literature on the anatomy of the caudal crural and plantar foot musculature of *Callithrix jacchus* and the data generated during the dissections. It is striking that the tibialis caudalis muscle is not mentioned in the consulted literature on *Callithrix jacchus* [21,23]. During the dissections, a previously non-described muscle was detected, which presented with identical origin, topography, and insertion to a muscle that is described in the anatomical works on the rhesus monkey [24,25,26]. Comparative anatomy allowed for its identification as the tibialis caudalis muscle.

Literature data show that the lumbrical muscles of the foot originate on the medial sides of the “flexor tendons” for digits III, IV, and V [21,23]. In contrast, the dissections revealed that not three but four lumbrical muscles of the foot are present in the common marmoset, with an additional lumbrical muscle originating on the medial side of the “flexor tendon” for the second digit. This situation is also present in the rhesus monkey [26].

The dissections revealed that the “flexor tendons” are composed of the individual tendons of the flexor digitorum medialis/longus muscle for digits I, II, and V, and the individual tendons of the flexor digitorum lateralis muscle for digits III and IV. Literature data confirm that the flexor digitorum medialis/longus muscle sends a tendon to the hallux [21,23]. It is stipulated that a tiny tendon branches off the medial side of the common tendon that gives origin to the individual tendons for digits II and V [21]. This tendon is considered the sole long tendon of the hallux [21]. Such a long tendon was observed during the dissections, but it did not proximally fuse with the medial side of the common tendon. On the contrary, it ran through the bifurcation of the common tendon into the individual tendons for digits II and V. Its muscle belly arose at the lateroplantar side of the calcaneus. Since this muscle is obviously shorter than the muscle that starts on the caudal side of the proximal aspect of the tibia, giving origin to the long common that splits into two individual tendons at the level of the proximal metatarsus, the flexor digitorum medialis/longus muscle consists of a long and a short part. The *m. flexor digitorum medialis*/*longus muscle pars longa* flexes digits II and V, while the *m. flexor digitorum medialis*/*longus muscle pars brevis* is responsible for flexion of the hallux.

Flexion of the hallux is further substantiated by the action of the flexor digiti primi/hallucis brevis muscle. At least, this is what the dissections demonstrated. This muscle is not mentioned in the anatomical literature on the common marmoset [21,23]. In contrast, it is comprehensively described in the rhesus monkey, in which it is composed of a medial and a lateral head [24,25,26]. This two-headed configuration was also encountered during the dissections of the *Callithrix jacchus* feet.

Further elaborating on the hallucal musculature, it should be noted that the adductor digiti primi/hallucis muscle is briefly described by Beattie, who mentions that only the transverse head is present, and no oblique head can be recognized in the common marmoset. More details are unfortunately missing [21]. Although a transverse and an oblique head are described in *Macaca mulatta* [24,25,26], the dissections in the common marmoset could only identify a muscle belly with an orientation and topography that paralleled those of the transverse head in the rhesus monkey. The dissections demonstrated that, in accordance with the observations made by Beattie, no oblique head of the adductor digiti primi/hallucis muscle exists in *Callithrix jacchus*.

At the opposite side of the foot sits the abductor digiti quinti/minimi muscle. In the consulted literature on the foot musculature of *Callithrix jacchus*, it is merely described as an easily recognizable muscular mass on the calcaneal tuber [21,23]. Unfortunately, further morphological or functional details are missing in the available sources. Again, the correct identification of this muscle was feasible by the comparison with its descriptions in the anatomical works on the foot musculature of the rhesus monkey [24,25,26]. Two other muscles with influence on the fifth digit, i.e., the abductor metatarsi quinti muscle and the flexor digiti quinti/minimi (brevis) muscle, also had to be identified by means of the available descriptions of these muscles in the rhesus monkey anatomical literature [24,25,26]. No information on these muscles could be found in the anatomical literature on the common marmoset’s foot musculature. The same is true for the contrahentes muscles of the foot.

In contrast, the interosseous muscles of the foot are mentioned in the consulted works on the foot musculature of *Callithrix jacchus*. However, particularities are not provided for the foot, as reference is made to the interosseous muscles of the hand [21]. The correct identification of the individual interosseous muscles was based on the data available for the rhesus monkey [24,25,26] and humans [27,28]. The organization of the pedal interosseous muscles into a lateral and a medial group, which both consist of a number of dorsal interosseous muscles (four in total) and plantar interosseous muscles (three in total), is a common trait of humans and the here-discussed primate species.

By including the muscles or muscle parts that are present in the rhesus monkey but not in the common marmoset in Table 2, and, vice versa, by indicating which muscles are typical for the common marmoset and cannot be recognized in the rhesus monkey, the foot musculature of both non-human primate species can easily be compared. It is interesting to see that the foot musculature of the common marmoset is very similar to that of the rhesus monkey. The quadratus plantae muscle (*m. quadratus plantae*, both in the N.A.V. and the T.A.2) is absent in the common marmoset, but present in the rhesus monkey [24,25,26]. In the rhesus monkey, this muscle flexes digits II to V [26]. These digits, albeit to various extents, are also flexed by the flexor digitorum lateralis muscle, the flexor digiti quinti/minimi (brevis) muscle, the flexor digitorum brevis muscle, and the flexor digitorum medialis/longus muscle. As a result, there seems to be no need for an accessory flexor muscle, and hence, we have the synonym for this muscle, i.e., flexor accessorius muscle (*m. flexor accessorius* in the T.A.2) [30]. As discussed above, the fibularis digiti quarti muscle is absent in the rhesus monkey, but present in the common marmoset. The functional relevance of possessing an additional muscle that can extend the fourth toe seems rather trivial. However, the accessory function of this muscle, i.e., the abduction of the fourth digit, could be meaningful for gripping the foot on smaller branches. Finally, it was observed that the common marmoset lacks the oblique head of the adductor digiti primi/hallucis muscle. At first sight, this is surprising in light of walking on thin branches. In the rhesus monkey, the oblique head allows for the opposition of the hallux as it originates from the bases of the second and third metatarsal bones and inserts into the axial side of the head of the first metatarsal bone and the proximal phalanx of the hallux. Such opposition, therefore, seems not to be possible in the common marmoset. Nevertheless, the orientation of the transverse head is well-suited to perform strong adduction of the hallux, which might be of the utmost importance while balancing on thin arboreal supports.

Performing functional or biomechanical analyses was beyond the scope of this anatomical work. The obtained anatomical data pose a prerequisite for future functional studies in which the question of how the muscle structures support the marmoset’s unique arboreal lifestyle could be answered. For example, muscle fiber length, relative physiological cross-sectional area, and relative muscle mass could be determined [36]. Herein lies an important and extensive study field to be explored, as functional adaptations of muscles to locomotor behavior in non-human primates have only seldom been described [37]. Another limitation of this study might be the small sample size. It should be noted that dissecting only four common marmosets may not be sufficient to capture the full range of any potential anatomical variations within this species. As no individual variations were observed within these four animals, the small sample size does not seem to limit the interpretation or generalization of the results. If variations are present in this species, they are statistically expected to occur rather infrequently. However, a follow-up study in which a 10-fold larger sample is investigated might bring clarity to this speculative matter. Such a study could potentially also reveal whether sex-based differences in the musculature of the common marmoset occur. As mentioned here, no anatomical variations were encountered in the small sample of four common marmosets, and in line with this, no differences could be observed between the males and the females. Future studies with larger sample sizes could target the questions of whether the absolute size, the cross-section of the muscle fibers, and the fiber type composition of the hind limb muscles differ between male and female common marmosets. To the best of the authors’ knowledge, no differences are described between men and women regarding the presence or absence of specific leg muscles. This is therefore not expected in the common marmoset.

## 5. Conclusions

This anatomical work provides a detailed textual description of the crural and foot musculature of the common marmoset that is supported by several color photographs arranged in multi-panel figures. Since the subsequent steps of the dissections are depicted, visualizing the muscles from the superficial to the deepest plane, it could be valuable as a dissection guide and anatomical atlas. Its use might be of particular interest when medical interventions have to be performed. Furthermore, planned anatomical studies, e.g., on the joint anatomy of the hind limb of *Callithrix jacchus*, could benefit from the comprehensive descriptions of the origins and insertions of the various muscles. Such an arthrological study would complement the available anatomical data on the locomotor system of the common marmoset [19].

During the dissections, it became clear that the available anatomical works on *Callithrix jacchus* were deficient in their descriptions of several muscles. For example, the short part of the flexor digitorum medialis/longus muscle is not yet described, only three and not four lumbrical muscles of the foot are defined in the available literature, and for information on the interosseous muscles of the foot, reference is made to those of the hand. Moreover, a few muscles were not mentioned at all. These include the extensor digitorum et digiti primi/hallucis brevis muscle, the fibularis digiti quarti muscle, the tibialis caudalis muscle, the flexor digiti primi/hallucis brevis muscle, the abductor metatarsi quinti muscle, the flexor digiti quinti/minimi (brevis) muscle, and the contrahentes muscles of the foot.

Therefore, the literature on rhesus monkey anatomy was consulted during the dissections, together with renowned human anatomy atlases. The foot musculature of the common marmoset largely paralleled that of the rhesus monkey. The few major differences that are emphasized in the Discussion section should be considered by veterinarians responsible for providing medical attention to marmosets or researchers working with marmosets.

## Figures and Tables

**Table 1:** Origin, insertion, and function of the interosseous muscles of the foot.

Muscle	Origin	Insertion	Function
15^I^: *m. interosseus plantaris I*/*primus* (medial group)	Plantar side basis os metatarsale II/secundum	Axial side basis proximal phalanx digitus II	Adduction digitus II Flexion digiti II, II, IV and V
15^II^: *m. interosseus plantaris II*/*secundus* (lateral group)	Plantar side basis os metatarsale IV/quartum and V/quintum	Axial side basis proximal phalanx digitus IV	Adduction digitus IV Flexion digiti II, II, IV and V
15^III^: *m. interosseus plantaris III*/*tertius* (lateral group)	Plantar side basis os metatarsale IV/quartum and V/quintum	Axial side basis proximal phalanx digitus V	Adduction digitus V Flexion digiti II, II, IV and V
16^I^: *m. interosseus pedis dorsalis I*/*primus* (medial group)	Plantar side basis os metatarsale II/secundum	Abaxial side basis proximal phalanx digitus II	Abduction digitus II Flexion digiti II, II, IV and V
16^II^: *m. interosseus pedis dorsalis II*/*secundus* (medial group)	Plantar side basis os metatarsale II/secundum	Abaxial side basis proximal phalanx digitus III	Abduction digitus III Flexion digiti II, II, IV and V
16^III^: *m. interosseus pedis dorsalis III*/*tertius* (lateral group)	Plantar side basis os metatarsale IV/quartum and V/quintum	Axial side basis proximal phalanx digitus III	Abduction digitus IV Flexion digiti II, II, IV and V
16^IV^: *m. interosseus pedis dorsalis IV*/*quartus* (lateral group)	Plantar side basis os metatarsale IV/quartum and V/quintum	Abaxial side basis proximal phalanx digitus IV	Abduction digitus IV Flexion digiti II, II, IV and V

**Table 2:** List of the muscles of the common marmoset foot with indication of the origin, insertion, and action, in alphabetical order. When a muscle is absent in the rhesus monkey, this is mentioned in brackets after the name of that muscle. In contrast, muscles or muscle parts that are present in the rhesus monkey but lacking in the common marmoset are listed as well.

Muscle	Origin	Insertion	Action
*m. abductor digiti primi*/*hallucis*	Medioplantar side calcanean tuber	Abaxial, proximal phalanx of digitus I	Abduction (and flexion) of digitus I
*m. abductor digiti quinti*/*minimi manus*	Calcanean tuber	Abaxial, proximal phalanx of digitus V	Abduction of digitus V
*m. abductor metatarsi quinti*	Calcanean tuber	Tuberosity of 5th metatarsal bone	Abduction of digitus V
*m. adductor digiti primi*/*hallucis*	*Caput transversum*: heads of 2nd and 3rd metatarsal bones, and contrahentes muscles II and IV *Caput obliquum additionally present in the rhesus monkey*	Axial, head of metatarsal bone I, and proximal phalanx digitus I	Adduction of digitus I
*mm. contrahentes digitorum pedis*	Common aponeurosis at the tendon of the long fibular muscle	Axial side, proximal phalanx of digiti II, IV and V	Adduction (with flexion) of digiti II, IV, and V towards the axis in between digiti III and IV
*m. extensor digiti primi*/*hallucis longus*	Crural interosseous membrane	Dorsal, distal phalanx of digitus I	Extension of digitus I, and dorsiflexion of the foot
*m. extensor digitorum et digiti primi*/*hallucis brevis*	Dorsolateral aspect of the calcaneal tuberosity	Dorsolateral, proximal phalanx of digitus I, and distal phalanges of digiti II to IV	Extension of digiti I to IV
*m. extensor digitorum longus*	Lateral tibial condyle, crural interosseous membrane and fibular head	Middle and distal phalanges of digiti II to V	Extension of digiti II to V
*m. fibularis brevis*	Distal two-thirds fibular diaphysis	Tuberosity of the 5th metatarsal bone	Eversion of the foot, and extension of the ankle (plantar flexion)
*m. fibularis digiti quarti* (absent in the rhesus monkey)	Proximal part of the caudolateral fibular margin	Abaxial, distal phalanx of digitus IV	Extension (and abduction) of digitus IV
*m. fibularis digiti quinti*/*minimi*	Proximal part of the caudolateral fibular margin	Abaxial, distal phalanx of digitus V	Extension (and abduction) of digitus V
*m. fibularis longus*	Proximal half of the fibular diaphysis, and fibular head	Abaxioplantar aspect of the basis of the 1st metatarsal bone	Eversion of the foot, extension of the ankle (plantar flexion), and opposition of digitus I
*m. flexor digiti primi*/*hallucis brevis*	*Caput mediale*: central and first tarsal bone. *Caput laterale*: first tarsal bone	*Caput mediale*: abaxial, proximal phalanx digitus I. *Caput laterale*: axial, proximal phalanx digitus I	Flexion of digitus I
*m. flexor digitorum lateralis*	Caudomedial side of the fibula and the interosseous membrane	Plantar, distal phalanges of digiti III and IV	Flexion of digiti III and IV
*m. flexor digiti quinti*/*minimi (brevis)*	5th metatarsal bone, and the tendon of the *m. fibularis longus* at this level	Abaxial side, proximal phalanx of digitus V	Flexion of digitus V
*m. flexor digitorum brevis*	*Caput superficiale*: caudomedial aspect of the calcanean tuber *Caput profundum*: tendon of m. flexor digitorum medialis/longus and the m. flexor digitorum fibularis	*Caput superficiale*: plantar side middle phalanx of digitus II. *Caput profundum*: plantar side middle phalanges of digiti III to V	Flexion of digiti II to V
*m. flexor digitorum medialis/longus*	*Pars longa:* caudal, middle third of the tibial shaft *Pars brevis:* lateroplantar side of the calcaneus	*Pars longa:* plantar, distal phalanges of digiti II to V *Pars brevis:* plantar, distal phalanx of digitus I	Flexion of digiti I, II and V
*m. gastrocnemius*	Caudal, proximal to lateral and medial femoral condyle (*caput laterale* and *caput mediale*, respectively)	Calcanean tuber	Extension of the ankle (plantar flexion) (and flexion of the knee)
*mm. interossei pedis dorsales*	Plantar surfaces of the bases of the 2nd, 4th, and 5th metatarsal bones	Proximal phalanges of digiti II (abaxial), III (axial and abaxial), and IV (abaxial)	Abduction (with flexion) of digiti II, III, and IV from the axis in between digiti III and IV
*mm. interossei plantares*	Plantar surfaces of the bases of the 2nd, 4th, and 5th metatarsal bones	Axial side, proximal phalanx of digiti II, IV, and V	Adduction (with flexion) of digiti II, IV, and V towards the axis in between digiti III and IV
*mm. lumbricales pedis*	Medial sides of the flexor tendons	Medioplantair, bases of the proximal phalanges and the metatarsophalangeal joints	Extension of the digits
*m. plantaris*	Caudal, proximal to the lateral femoral condyle	Plantar aponeurosis	Extension of the ankle (plantar flexion) (and flexion of the knee) through tension on the plantar aponeurosis
*m. quadratus plantae*: absent in the common marmoset, but present in the rhesus monkey	/	/	/
*m. soleus*	Caudal aspect of fibular head	Calcanean tuber	Extension of the ankle (plantar flexion)
*m. tibialis caudalis*	Proximal half of caudal side tibia, interosseous membrane, medial side fibula	Plantar, bases of metatarsal bones II to IV	Extension of the ankle (plantar flexion) and inversion of the foot
*m. tibialis cranialis*	Lateral condyle of the tibia and proximal two-thirds of its shaft	Medial belly: medial side of the 1st tarsal bone; Lateral belly: head of the 1st metatarsal bone	Dorsiflexion of the ankle, inversion of the foot, and abduction of digitus I

## Data Availability

All data generated during the project are presented in the manuscript.
